# Growth-Promoting Effects and Mechanisms of Synthetic Plant Growth-Promoting Rhizobacteria on Maize Seedlings

**DOI:** 10.3390/microorganisms13112460

**Published:** 2025-10-28

**Authors:** Shuang Yu, Minlong Mao, Hengfei Zhang, Huanyu Song, Yu Sun

**Affiliations:** 1 College of Life Sciences and Technology, Mudanjiang Normal University, Mudanjiang 157011, China; 2 State Key Laboratory of Black Soils Conservation and Utilization, Northeast Institute of Geography and Agroecology, Chinese Academy of Sciences, Changchun 130102, China; 3 Key Laboratory of Straw Comprehensive Utilization and Black Soil, Conservation College of Life Science, The Ministry of Education, Jilin Agricultural University, Changchun 130118, China

**Keywords:** maize, PGPR, SynComs, RNA-seq, promoting mechanism

## Abstract

With the development of microbial fertilizers, efforts have been made to enrich the strain resources of plant growth-promoting rhizobacteria (PGPR) in maize and to compare the growth-promoting effects of synthetic microbial communities (SynComs) with those of single strains. To achieve this, phenotypic measurements and RNA sequencing (RNA-seq) were performed on maize roots treated with SynComs and single-strain bacterial suspensions, aiming to investigate the regulatory influence of PGPR on differential gene expression and key metabolic pathways in maize roots. In this study, 59 PGPR strains were selected, representing genera including *Bacillus*, *Pseudomonas*, *Burkholderia* sp., *Curtobacterium pusillum*, *Acidovorax*, *Sphingobium*, *Mitsuaria*, *Bacterium*, *Rhodanobacter*, *Variovorax*, *Ralstonia*, *Brevibacillus*, *Terrabacter*, *Flavobacterium*, *Comamonadaceae*, *Achromobacter*, *Paraburkholderia*, and *Massilia*. Based on the growth-promoting effects observed in pot experiments, optimal bacterial strains were selected according to the principles of functional complementarity and functional superposition to construct the SynCom. The selected strains included *Burkholderia* sp. A2, *Pseudomonas* sp. C9, *Curtobacterium pusillum* E2, and *Bacillus velezensis* F3. The results demonstrated that individual strains exerted measurable growth-promoting effects on seedlings; however, the growth-promoting capability of the SynCom was significantly stronger than that of single strains. The synthetic microbial community ALL group markedly increased root length, shoot fresh weight, shoot dry weight, number of branches, and number of root tips in maize seedlings. RNA-seq analysis of maize roots treated with the SynCom (ALL group) was conducted in comparison with CK, A2, C9, E2, and F3 treatment groups. A total of 5245 differentially expressed genes (DEGs) were identified, of which only 133 were common across treatments. GO and KEGG analyses revealed that DEGs were enriched in multiple biological processes, including cellular amide biosynthetic and metabolic processes, flavonoid biosynthetic and metabolic processes, carbohydrate metabolism, amino acid metabolism, lipid metabolism, and translation. The majority of enriched pathways were associated with primary and secondary metabolism, indicating that these bacterial strains promote plant growth by modulating a wide range of metabolic pathways in plant cells. Overall, this study provides a molecular framework for understanding the mechanisms underlying the growth-promoting effects of SynComs on maize roots and offers valuable insights for future research aimed at identifying key regulatory genes.

## 1. Introduction

Maize (*Zea mays* L.) is an annual herb belonging to the genus *Zea* L. of the Gramineae family (Poaceae Barnhart). It is a major food crop in many parts of the world, ranking as the third most important food crop in China after wheat and rice [1]. Maize has a wide range of applications, diverse modes of utilization, and numerous varieties. Among these, approximately 90% of maize is used for silage and industrial processing, while the remaining 10% is consumed directly by humans [2]. When used as silage feed, maize demonstrates several advantages over other silage crops, including high biomass yield, elevated starch content, favorable fermentation properties, and strong adaptability [3]. In contrast, maize grown for fresh consumption emphasizes ear harvest, with types including sweet maize, waxy maize, and sweet-waxy maize. Owing to its relatively high content of nutrients such as protein, crude fat, and starch, as well as its provision of essential fatty acids and amino acids, fresh maize has gained considerable consumer attention [4,5]. Beyond the ear, various maize by-products also possess medicinal properties. For example, maize stalks exhibit antioxidant and hypoglycemic effects [6]; maize cobs have been shown to lower blood sugar and regulate lipid metabolism [7]; and maize bracts and maize silk contribute to reducing blood lipid levels [8]. Therefore, enhancing the yields of all maize types plays an important role in strengthening national grain reserves, stimulating economic development, and improving public health. By 2024, the total maize planting area in China reached 44,700,000 hectares, with an output of 294.67 billion kg, reflecting significant improvements compared with previous years [9].

The maize growth cycle requires relatively high levels of nutrient elements. Thus, the application of chemical fertilizers has been a critical measure to increase maize yield. However, prolonged and excessive fertilizer use has led to soil degradation issues, including reduced soil organic matter, erosion of the tillage layer, and compaction [10]. To mitigate ecological and environmental problems associated with long-term chemical fertilizer application, pollution-free, non-toxic, harmless, and naturally derived microbial fertilizers have been developed and increasingly applied [11].

Plant growth-promoting rhizobacteria (PGPR), as a widely used category of microbial fertilizers, play a crucial role in supporting plant health and regulating the soil microbial ecological environment [12]. Numerous studies have demonstrated that PGPR contribute to nitrogen fixation, phosphorus solubilization, potassium solubilization, antibiotic secretion, and hormone synthesis [13]. In addition, PGPR colonize plant roots, enhance nutrient solubilization, and thereby improve the absorption and utilization of mineral nutrients by plants [14]. Research to date has confirmed the growth-promoting effects of bacteria such as *Bacillus* and *Pseudomonas* [15]. However, single strains often present limitations, including incomplete fermentation capacity and reduced quality of microbial fertilizers. In studies on lignocellulose conversion, the required enzymes for degrading complex substrates have been found to be distributed across multiple strains, which cooperate in the conversion process through complementary sub-functions [16]. Similarly, research on walnut seedling growth has revealed that the poor performance of single strains in field conditions is due to competition with native microorganisms [17]. Against this backdrop, researchers have increasingly shifted from relying on single strains to developing synthetic microbial communities.

Synthetic microbial communities (SynComs) are designed mixtures of selected strains that aim to improve the stability of microbial communities beneficial to plants through synergistic interactions. This approach enables detailed evaluation of host–microbe and microbe–microbe interactions under controlled and reproducible conditions [18]. Compared with individual PGPR strains, SynComs exhibit stronger resilience, greater stability, and enhanced capacity to increase plant resistance to environmental stressors [19,20]. Inoculation of SynComs into seedling substrates and soils has been shown to more effectively promote crop growth by broadening plant metabolite profiles and enhancing microbial community assembly [21]. However, studies investigating the plant growth-promoting effects of SynComs in the maize rhizosphere remain limited, and mechanistic insights into how SynComs promote maize growth are even less developed.

Currently, most plant growth-promotion experiments aim to elucidate growth-promotion mechanisms through methods such as indole-3-acetic acid (IAA) production, phosphate solubilization, potassium release, nitrogen fixation, and analysis of microbial community composition [22,23]. Some studies also employ metabolomics to conduct differential analysis of plant-produced metabolites [24]. These approaches primarily emphasize the detailed characterization of biochemical processes, while relatively fewer studies focus on underlying molecular mechanisms. Because gene expression dynamically responds to different stimuli over time, RNA-seq has demonstrated strong potential for analyzing gene expression in plant systems [25]. In maize research, RNA-seq has been widely applied and has become a powerful tool for identifying differentially expressed genes (DEGs) [26,27]. Nevertheless, research on the growth-promoting effects and mechanisms of synthetic microbial communities (SynComs) in maize seedlings remains limited, and few studies have explained the growth-promotion mechanisms of plant growth-promoting rhizobacteria (PGPR) in maize from a molecular perspective [28].

In this study, rhizosphere soil from Kuancheng District, Changchun City, was used as the test material to screen and verify PGPR with varying growth-promoting capacities and to evaluate their effects on maize. PGPR with strong growth-promoting potential were selected to construct SynComs. The growth-promoting effects of single strains and SynComs were compared, and RNA-seq analysis was employed to investigate the underlying mechanisms. These results provide a theoretical framework and technical support for the development and application of specialized microbial fertilizers in maize cultivation.

## 2. Materials and Methods

### 2.1. Isolation and Selection of Functional Strains

The experimental research was conducted in Changchun City, Jilin Province, at the Northeast Institute of Geography and Agroecology, Chinese Academy of Sciences (41°2′24″ N, 125°24′36″ E). After weighing an appropriate amount of soil, impurities such as roots were removed, and the sample was transferred to a centrifuge tube and stored at −80 °C for later use.

One gram of maize rhizosphere soil was weighed, and a soil suspension was prepared in an Erlenmeyer flask under sterile conditions. The suspension was shake-cultured at 140 r/min for 20 min at 25 °C. After gradient dilution, soil suspensions at different concentrations (10^−3^, 10^−4^, 10^−5^, 10^−6^, 10^−7^, 10^−8^, 10^−9^) were spread onto Pikovskaya’s agar medium, Alexandrov agar medium, Ashby agar medium, and Endophytic diazotrophs medium. The plates were incubated at 30 °C. Single colonies with obvious transparent zones were selected, repeatedly purified, and subcultured multiple times prior to preservation. Isolated and purified colonies were inoculated into the corresponding selective liquid media and cultured in a constant-temperature shaker at 120 r/min and 30 °C for 24 h. Once the liquid medium became turbid, 1 mL of bacterial suspension and 1 mL of 80% glycerol were simultaneously added to the 2 mL centrifuge tube. Tubes were subsequently stored at −80 °C for long-term preservation.

Subsequently, 1 mL of bacterial suspension was placed into a 1.5 mL centrifuge tube and submitted to Sangon Biotech Co., Ltd. (Changchun, China) for sequencing. During the sequencing process, the V1-V9 hypervariable region of the bacterial 16S rRNA gene was amplified by PCR using the primer pair 27F (5′-AGAGTTTGATCMTGGCTCAG-3′) and 1492R (5′-GGTTACCTTGTTACGACTT-3′). The resulting sequences were analyzed by alignment in the NCBI database to determine the taxonomic identity of the strains.

### 2.2. Promoting Effect of a Single Strain on Maize Seedlings

First, 59 plant growth-promoting rhizobacteria (PGPR) preserved in 80% glycerol were activated on 50% tryptic soy broth (TSB) solid medium. If contaminants appeared among the activated strains, an inoculation loop was used to pick different colonies and streak them separately on 50% TSB solid plates for isolation and purification. The selected strains were subsequently cultured in liquid medium for 24 h. One milliliter of bacterial suspension was submitted for 16S sequencing, and the obtained results were compared in the NCBI database. Strains with consistent sequencing results were preserved in 80% glycerol. According to the result of the NCBI database, a phylogenetic tree was constructed with MEGA11 according to strain classification.

Maize seeds were surface-sterilized by soaking in 70% ethanol for 3–6 min, followed by rinsing with sterile distilled water at least three times [29]. Using sterile tweezers, seeds were placed with the embryo facing upward on filter paper in a plastic box. The box was covered and incubated in the dark at 30 °C for 72–96 h. Sequenced strains were re-cultured in liquid medium with shaking, and bacterial suspensions were centrifuged at 5000 r/min. The supernatant was discarded, and the pellet was resuspended in sterile 0.85% saline. This washing process was repeated three times. The resulting bacterial suspension was adjusted by measuring OD600 with a multifunctional enzyme marker, diluted to OD600 ≈ 0.5, and then further diluted with sterile water at a ratio of 1:100. The diluted suspension was added to sterilized vermiculite at a ratio of 1 mL:1 g. Hoagland’s formula hydroponic nutrient solution A and solution B were then added at a water-to-solution ratio of 500:1. The mixture was stirred thoroughly and left to stand for 12 h.

For planting, 100 mL tin cans were filled with 40–50 mL of water and sterilized. Disposable plastic cups (~350 mL) were used as planting pots, with holes punched at the bottom. Gauze strips were threaded through the base of the pots and extended into the tin cans to ensure water absorption by capillarity. Sixty grams of vermiculite treated with the bacterial suspension were placed into each plastic pot. Germinated maize seeds with roots 2–3 cm in length were transplanted into the vermiculite with their roots buried. Based on the strain activation sequence, a total of six experimental batches were established, each containing 9–13 treatment groups and 1 control group, with six replicates per group. The treatment group refers to the group with strains added, and the control group did not add any strains.

The growth status of maize seedlings and vermiculite humidity were observed daily. If the humidity was excessive, pots were removed from the water to allow water isolation treatment. Samples were collected 10–15 days after planting. For each treatment, three uniformly growing maize plants were selected. After the roots were cleaned, the shoots and roots were separated. Root systems were scanned with a Root-zone Scanner (WinRHIZO, REGENT, Quebec, QC, Canada) to determine root length, number of root tips, and number of bifurcations. For shoots, fresh weight and plant height were measured, followed by drying in an oven at 80 °C until constant weight was achieved to record dry weight. The remaining three maize plants were labeled, frozen in liquid nitrogen, and stored at −80 °C for later use.

### 2.3. Promoting Effect of SynComs on Maize Seedlings

**Figure 1:**
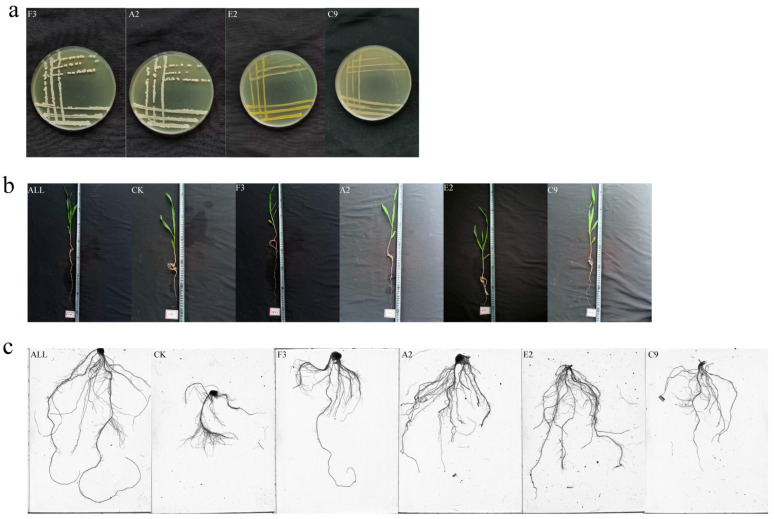
Growth status of strains and maize seedlings treated with synthetic microbial communities. (**a**) Strain morphology; (**b**) seedling morphology; (**c**) root system scanning image.

Six growth indicators of maize seedlings were comprehensively analyzed: plant height, fresh weight, dry weight, root length, number of root tips, and number of branches. Based on the principle of complementary functional characteristics, the selected strains were classified into three categories: (i) strains promoting both root and shoot growth, (ii) strains promoting root growth, and (iii) strains promoting shoot growth. The four strains selected were *Burkholderia* sp. A2, *Pseudomonas* sp. C9, *Curtobacterium pusillum* E2, and *Bacillus velezensis* F3. These strains were combined to construct a synthetic microbial community (SynCom). The strain composition is presented in Table 1, and representative colony morphologies are shown in Figure 1a.

The selected strains A2, C9, E2, and F3 were activated and liquid-cultured following the activation method for single strains. Equal volumes of the four bacterial suspensions were combined in the ALL treatment group to prepare a synthetic bacterial suspension. The OD600 of the suspension was measured using a multifunctional enzyme marker and adjusted to approximately 0.5. The suspension was then diluted with sterile water at a ratio of 1:100. The diluted suspension was added to sterilized vermiculite at a ratio of 1 mL:1 g. Hoagland’s formula hydroponic nutrient solution A and solution B were each added at a water-to-solution ratio of 500:1. After thorough mixing, the medium was left to stand for 12 h.

Following the planting method used for single strains, maize seeds were sown into vermiculite. A total of five treatment groups and one control group were established, each with 12 replicates. The five treatment groups was F3, A2, E2, C9, and ALL(syncoms). The growth of maize seedlings and the humidity of vermiculite were monitored daily. If the humidity was excessive, pots were removed from the water surface for water isolation treatment. Samples were collected after 14 days of growth. For each treatment, six uniformly growing maize plants were selected. Roots were thoroughly cleaned, and the above-ground and below-ground parts were separated. Representative images of plants are shown in Figure 1b. Root systems were scanned using a Root-zone Scanner (WinRHIZO, REGENT, Canada) to determine root length, number of root tips, and number of forks; a representative scanned image is shown in Figure 1c. For shoots, fresh weight and plant height were measured, after which the samples were dried in an oven at 80 °C until constant weight was achieved to record dry weight. The remaining six maize plants were labeled, frozen in liquid nitrogen, and stored at −80 °C for later use.

### 2.4. RNA-Seq Analysis of Maize Seedling Roots Under Treatments with Different Strains

#### 2.4.1. RNA Extraction and cDNA Library Construction

Based on the above experimental results, maize plants from treatment groups CK, F3, A2, E2, C9, and ALL in the synthetic microbial community (SynCom) experiment were selected. From each group, 5–6 g of maize roots were washed with clean water, and three biological replicates were prepared. Root samples were placed into 10 mL centrifuge tubes and immediately frozen in liquid nitrogen for storage. Total RNA was extracted from root tissues using the RNAPrep Pure Plant Kit (DP432, TIANGEN, Beijing, China). RNA integrity was assessed with the Agilent 2100 Bioanalyzer. Qualified RNA samples were used to enrich mRNA containing poly(A) tails via Oligo(dT) magnetic beads. RNA was then fragmented to ~300 bp in length by ion fragmentation. A cDNA library was constructed by synthesizing the first strand of cDNA with 6-base random primers and reverse transcriptase using RNA as the template, followed by synthesis of the second strand of cDNA using the first strand as the template. Library quality was evaluated with the Agilent 2100 Bioanalyzer. Paired-end (PE) sequencing was subsequently performed on the Illumina-MiSeq platform (Illumina, San Diego, CA, USA) at the Shanghai Personal Biotechnology corporation, ensuring that the effective concentration of the library was ≥2 nM [30].

#### 2.4.2. Analysis of the Transcriptome Sequencing Data

RNA-seq was conducted by Personal Biotechnology Co., Ltd. (Shanghai, China). Sequencing generated image files that were processed into raw data by the Personal GenesCloud Platform (https://www.genescloud.cn/home (accessed on 10 June 2025)). The raw data were filtered to remove low-quality reads and adapter-containing sequences, resulting in clean reads. Clean reads were aligned to the reference genome using HISAT2 (http://ccb.jhu.edu/software/hisat2/index.shtml (accessed on 10 June 2025)) [31]. During HISAT2 alignment, default parameters were applied for non-strand-specific libraries, while strand-specific libraries required specifying the library type (i.e., –rna-strandness RF for the first-strand case and –rna-strandness FR for the second-strand case). Read counts for each gene were obtained using the Union scheme in HTSeq and served as raw gene expression levels. To allow comparison across genes and samples, expression levels were normalized using FPKM (fragments per kilobase of transcript per million mapped reads). Differential expression analysis was performed with DESeq2, applying thresholds of |log2FoldChange| > 1 and *p*-value < 0.05. Gene function annotation was carried out using the Gene Ontology (GO; http://geneontology.org (accessed on 10 June 2025)) and Kyoto Encyclopedia of Genes and Genomes (KEGG; http://www.kegg.jp/ (accessed on 10 June 2025)) databases. Differentially expressed genes (DEGs) were categorized based on their GO terms and KEGG pathways. P-values were calculated using the hypergeometric distribution method, with significant enrichment defined as *p*-value < 0.05. GO terms and KEGG pathways significantly enriched with DEGs compared to the genomic background were identified, thereby revealing the principal biological functions associated with these genes. Transcription factor (TF) analysis was performed by comparing gene sequences against the Plant Transcription Factor Database (PlantTFDB), allowing prediction of TFs and their corresponding families.

### 2.5. Statistical Analysis

In this experiment, differences between treatment and control groups were evaluated using a *t*-test, while one-way analysis of variance (ANOVA) with least significant difference (LSD) was applied to assess multiple comparisons. Graphs were generated using Origin 2021, with significance set at *p* < 0.05. Principal component analysis (PCA) and Venn diagram construction were performed using the *prcomp* package in R software (https://www.r-project.org/; R Core Team, Auckland, New Zealand; version released 1 January 2020 (accessed on 15 June 2025)) [32]. Clustering of gene expression was conducted using the R *pheatmap* package. Boxplots and volcano plots were generated with the *boxplot*() function from the base R package and the *ggplot2* package, respectively. All diagrams were subsequently refined using Adobe Illustrator CC 2018.

## 3. Results

### 3.1. Analysis of 16S rDNA Sequence of PGPR of Maize

**Figure 2:**
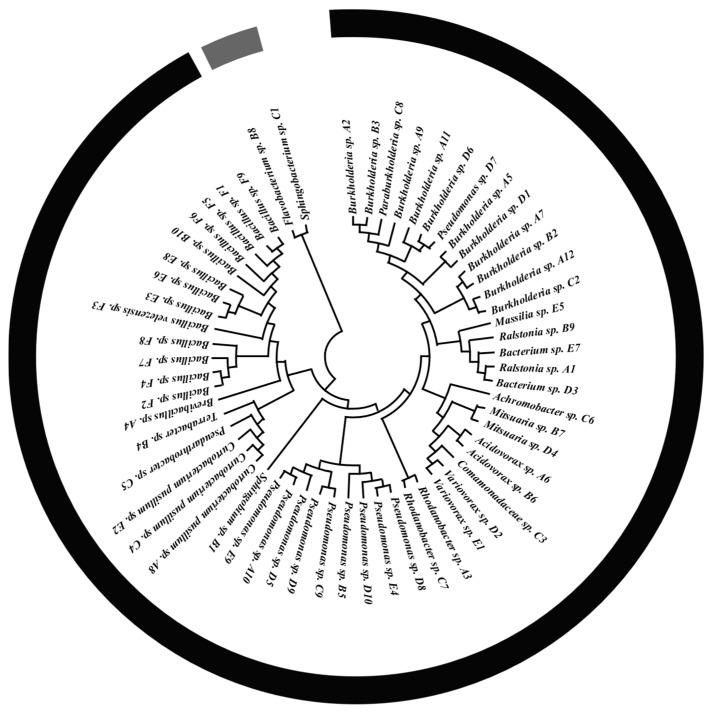
Phylogenetic tree of 59 bacterial strains constructed from 16S rDNA sequences.

A total of 59 plant growth-promoting bacteria were screened from maize rhizosphere soil, representing 19 genera: *Bacillus* (13 strains), *Pseudomonas* (11 strains), *Burkholderia* sp. (11 strains), *Curtobacterium pusillum* (3 strains), *Acidovorax* (2 strains), *Sphingobium* (2 strains), *Mitsuaria* (2 strains), *Bacterium* (2 strains), *Rhodanobacter* (2 strains), *Variovorax* (2 strains), *Ralstonia* (2 strains), *Brevibacillus* (1 strain), *Terrabacter* (1 strain), *Flavobacterium* (1 strain), *Comamonadaceae* (1 strain), *Achromobacter* (1 strain), *Paraburkholderia* (1 strain), and *Massilia* (1 strain). A phylogenetic tree was constructed using 16S rDNA sequences (Figure 2).

### 3.2. Evaluation of Growth-Promoting Ability of a Single PGPR

Fourteen-day-old maize seedlings grown in vermiculite were harvested, and fresh weight, dry weight, and plant height were measured. Root length, number of root tips, and number of branches were also analyzed. Five treatments (D4, D10, E4, E2, and E1) significantly increased plant height compared with the control. The remaining treatments showed differences relative to the control, but these were not significant. One treatment, E2, significantly increased the fresh weight of the shoots, while differences observed in the other treatments were not significant. Nine treatments (A2, D5, D2, D8, D10, D3, D4, C7, and A7) significantly increased the number of root tips. For the remaining treatments, differences relative to the control were observed but not significant. None of the treatments significantly increased dry weight, root length, or number of root forks, although some treatments showed non-significant differences compared with the control.

### 3.3. Evaluation of Growth-Promoting Ability of a SynComs

**Figure 3:**
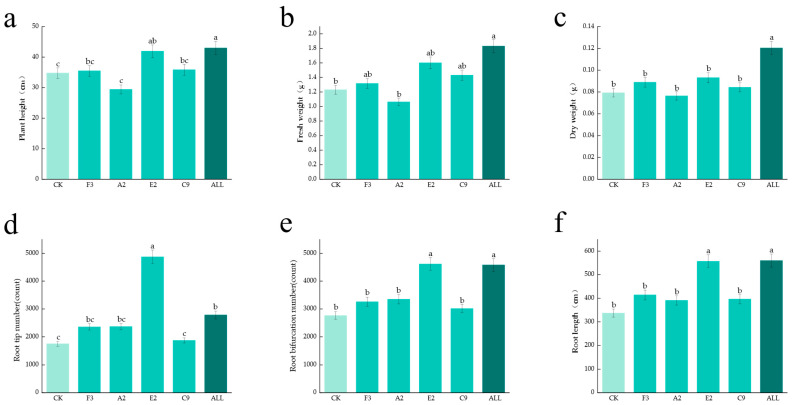
Growth-promoting effects of single strains and SynComs. (**a**) Maize plant height; (**b**) fresh weight of above-ground parts; (**c**) dry weight of above-ground parts; (**d**) number of root tips; (**e**) number of tillers; (**f**) root length.

Fourteen-day-old maize seedlings grown in vermiculite were harvested, and fresh weight, dry weight, and plant height were measured. Root length, number of root tips, and number of branches were also analyzed. As shown in Figure 3, data from all treatments in the ALL group were significantly higher than those in the CK group, and most values exceeded those observed in the single-strain treatment groups. These results demonstrate that SynComs prepared from multiple strains exhibit strong growth-promoting effects, with greater growth-promoting capacity than individual strains.

### 3.4. RNA-Seq Analysis of Maize Seedlings in Response to Strains

Transcriptome sequencing was performed on maize root systems, yielding raw transcriptome data from 24 samples. The raw data were subsequently filtered and screened. After removal of adapters and low-quality sequences, clean reads accounted for more than 97.05% of the original sequence data for each sample (Table 2).

### 3.5. Correlation Analysis and Sample Clustering Evaluation of Transcriptome Data

**Figure 4:**
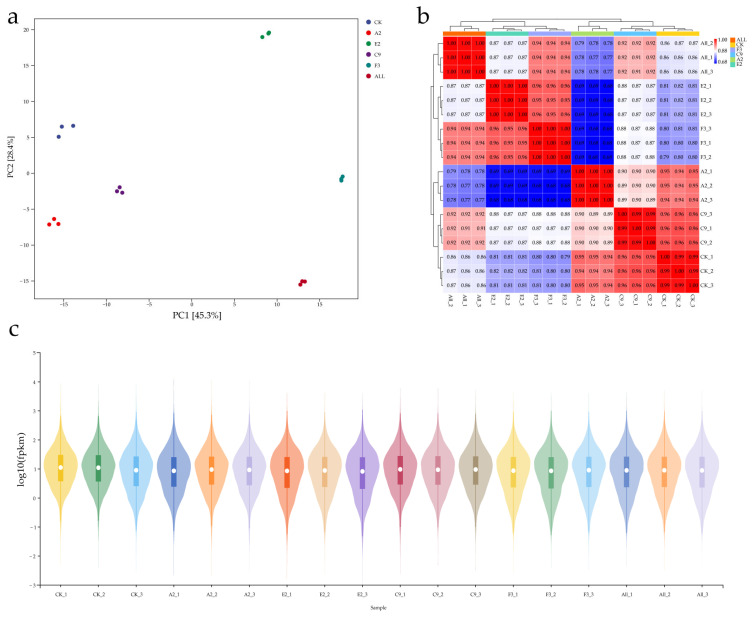
Quality analysis of transcriptome data from maize roots under different conditions. (**a**) Principal component analysis (PCA) plot showing sample clustering based on gene expression profiles; (**b**) heatmap of correlation analysis showing similarity among biological replicates in each group (ALL, CK, F3, A2, E2, C9); (**c**) boxplot presenting log10(FPKM) values for transcript abundance distribution across samples.

This experiment evaluated the reproducibility and distribution of transcriptome sequencing data using sample correlation analysis, gene expression boxplots, and principal component analysis (PCA). PCA analysis (Figure 4a) showed that PC1 (45.3%) and PC2 (28.4%) clustered samples from the same group together while clearly separating different groups. These results indicate good reproducibility within groups and significant differences between the experimental and control groups. This analysis confirms the rationality of sample grouping and the reliability of the sequencing data.

The sample correlation heatmap showed that correlation coefficients among biological replicates were all above 0.9, indicating high consistency in gene expression patterns (Figure 4b). Comparison of the SynComs group with other groups revealed that each group clustered within its own branch without strong internal associations, indicating significant transcriptomic differences among the groups.

To further evaluate data quality and comparability, boxplots of gene expression were generated (Figure 4c). The results showed that fragments per kilobase of exon per million mapped reads (FPKM) values were uniformly distributed on a log10 scale across samples, with highly similar medians and interquartile ranges. This uniform distribution demonstrates the effectiveness of data normalization, with no apparent outliers or extreme deviations. In addition, the overall distribution of FPKM values confirmed the absence of systematic bias in gene expression levels across samples, ensuring that the dataset was balanced and suitable for subsequent differential gene expression analysis.

### 3.6. Differentially Expressed Gene (DEGs) Analysis

To enable comparison of gene expression levels across different genes and samples, fragments per kilobase of transcript per million mapped reads (FPKM) were used as the measurement standard [33]. Differential expression analysis was performed using DESeq2, with screening conditions set as |log2 fold change| > 1 and *p* < 0.05. Statistical results showed that, compared with the CK group, the ALL group had 3861 differentially expressed genes (DEGs), including 2290 upregulated and 1571 downregulated. Compared with the F3 single-strain treatment, the ALL group had 1698 DEGs (791 upregulated and 907 downregulated). Compared with the A2 single-strain treatment, the ALL group had 3694 DEGs (1873 upregulated and 1821 downregulated). Compared with the E2 single-strain treatment, the ALL group had 3682 DEGs (1949 upregulated and 1733 downregulated). Compared with the C9 single-strain treatment, the ALL group had 2771 DEGs (1412 upregulated and 1359 downregulated). Compared with the NP single-strain treatment, the ALL group had 1698 DEGs (791 upregulated and 907 downregulated).

**Figure 5:**
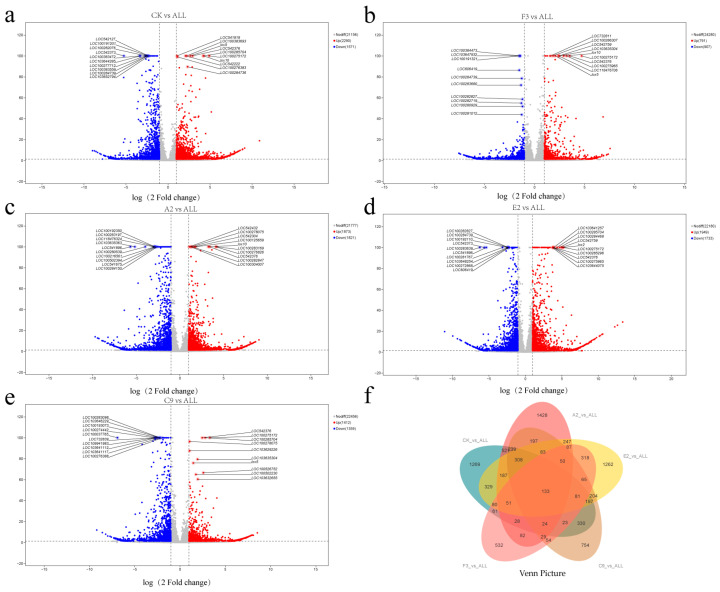
Volcano plots and Venn diagrams of differentially expressed genes (DEGs) from different strain comparison groups. (**a**) CK vs. ALL; (**b**) F3 vs. ALL; (**c**) A2 vs. ALL; (**d**) E2 vs. ALL; (**e**) C9 vs. ALL; (**f**) Venn diagram of DEGs across all groups.

The results indicated that the number of DEGs between the ALL group and each single-strain treatment was lower than CK groups. This may reflect the influence of strain-derived metabolites on root growth and development, with similar transcriptional effects observed between the ALL group and individual strains. In addition, except for F3, upregulated DEGs outnumbered downregulated ones, suggesting that different strain treatments may trigger comparable transcriptional responses. Comparisons of the ALL group with the A2 and E2 groups revealed relatively high numbers of DEGs, comparable to those observed against the CK group. This suggests that these two strains exert weaker effects on maize roots, leading to greater transcriptomic divergence from the ALL group. To more clearly distinguish common and unique DEGs, the DEGs from different comparisons were classified and summarized (Figure 5f). Across all comparisons, 5245 genes exhibited differential expression, of which only 133 were shared.

### 3.7. Gene Set Enrichment Analysis (GSEA)

To explore the biological functions of DEGs at different growth stages and the potential metabolic regulation mechanisms, GSEA of GO and KEGG pathways was performed on the transcriptome data of the synthetic microbial community group (ALL) and the single-strain treatment and control groups (CK, F3, A2, E2, C9) in this study. The comparison groups were designated as Group K (ALL vs. CK), Group F (ALL vs. F3), Group A (ALL vs. A2), Group E (ALL vs. E2), and Group C (ALL vs. C9).

#### 3.7.1. GO Functional Classification Analysis of Differentially Expressed Genes (DEGs)

**Figure 6:**
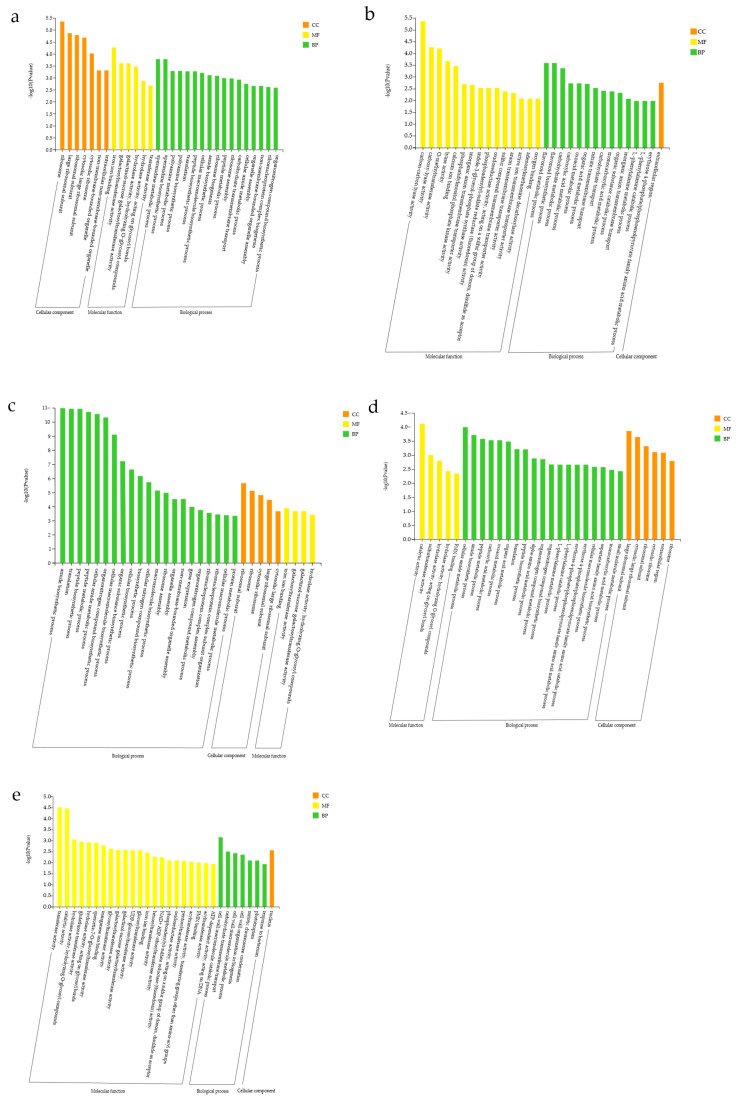
Comparison of GO functional annotation results for differentially expressed genes (DEGs) in treatment combinations. (**a**) CK vs. ALL; (**b**) F3 vs. ALL; (**c**) A2 vs. ALL; (**d**) E2 vs. ALL; (**e**) C9 vs. ALL.

The results of GO enrichment analysis comprised three categories: biological process (BP), cellular component (CC), and molecular function (MF). For Group K (ALL vs. CK), DEGs enriched in the CC category were mainly ribosome, large ribosomal subunit, ribosomal subunit, cytosolic large ribosomal subunit, cytosolic ribosome, non-membrane-bounded organelle, and intracellular non-membrane-bounded organelle. DEGs significantly enriched in CC pathways were all upregulated, indicating that, compared with CK, the ALL group exhibited greater activity of organelles involved in biosynthesis and transport, likely due to the accumulation and secretion of growth-promoting hormones and synthesized proteins via transmembrane transport. In MF, DEGs were significantly enriched in iron ion binding, galactosyltransferase activity, galactinol–sucrose galactosyltransferase activity, hydrolase activity (hydrolyzing O-glycosyl compounds and acting on glycosyl bonds), and transferase activity. These findings suggest that enzyme-catalyzed reactions play an important role in metabolic regulation. In BP, DEGs were significantly enriched in spermidine metabolic process, spermidine biosynthetic process, polyamine metabolic process, polyamine biosynthetic process, translation, peptide biosynthetic process, cellular macromolecule biosynthetic process, amide biosynthetic process, ribosome biogenesis, peptide metabolic process, ribosome assembly, carbohydrate transmembrane transport, cellular amide metabolic process, organelle assembly, non-membrane-bounded organelle assembly, ribonucleoprotein complex biogenesis, and organonitrogen compound biosynthetic process. Overall, DEGs were enriched primarily in metabolic processes and the biosynthesis of secondary metabolites, indicating that, compared with the control group, the ALL group may enhance DEG accumulation by altering metabolic activity. Further analysis showed that DEGs involved in peptide biosynthetic and metabolic processes were significantly upregulated, suggesting more active synthesis and metabolism of peptide compounds in the ALL group, which in turn supports plant growth (Figure 6a).

After comparing Group K, enrichment analysis was conducted between the ALL group and each single-strain group (F3, A2, E2, C9). In Group F (ALL vs. F3), DEGs showed low enrichment significance in CC, with only moderate enrichment in the extracellular region. This suggests that metabolic activities in the extracellular region differ in this group, which may potentially influence plant development. In MF, DEGs were significantly enriched in carbon–carbon lyase activity, carboxy-lyase activity, O-methyltransferase activity, lyase activity, calcium ion binding, phosphatidylinositol phosphate kinase activity, inorganic anion transmembrane transporter activity, indole-3-glycerol-phosphate synthase activity, phosphoadenylyl-sulfate reductase (thioredoxin) activity, oxidoreductase activity acting on sulfur group donors with disulfide as acceptor, sulfur compound transmembrane transporter activity, anion transmembrane transporter activity, active transmembrane transporter activity, adenosylmethionine decarboxylase activity, and oxygen binding. The enriched DEGs included various enzyme proteins, transmembrane transporters, and components related to signal transduction, indicating that plant biosynthesis is regulated through a complex signaling network, with significant differences observed between the ALL and F3 groups. In BP, DEGs were significantly enriched in flavonoid metabolic process, flavonoid biosynthetic process, carbohydrate metabolic process, carboxylic acid metabolic process, oxoacid metabolic process, organic acid metabolic process, nitrate transmembrane transport, carbohydrate transport, monocarboxylic acid metabolic process, organic substance catabolic process, inorganic anion transmembrane transport, L-phenylalanine metabolic process, L-phenylalanine catabolic process, and the erythrose-4-phosphate/phosphoenolpyruvate family amino acid metabolic process. Collectively, DEGs were still concentrated in various metabolic processes and secondary metabolite synthesis, suggesting that the ALL group may affect plant growth and development by altering metabolic activity (Figure 6b).

Enrichment analysis was also conducted for Group A (ALL vs. A2). In CC, DEGs were predominantly enriched in ribosomal subunit, ribosome, cytosolic ribosome, large ribosomal subunit, and cytosolic large ribosomal subunit. All of these DEGs were upregulated, indicating that compared with A2, the ALL group promotes protein synthesis by upregulating ribosome-related genes, thereby influencing plant growth and development. In MF, DEGs were significantly enriched in iron ion binding, galactosyltransferase activity, galactinol–sucrose galactosyltransferase activity, and hydrolase activity (hydrolyzing O-glycosyl compounds). This again involved enzyme proteins and functions related to signal transduction, but the number of DEGs in this category was small, suggesting limited differences in signal network regulation between the ALL and W24 groups. In BP, DEGs were significantly enriched in amide biosynthetic process, translation, peptide biosynthetic process, peptide metabolic process, cellular amide metabolic process, organonitrogen compound biosynthetic process, cellular macromolecule biosynthetic process, organic substance biosynthetic process, cellular biosynthetic process, biosynthetic process, cellular nitrogen compound biosynthetic process, macromolecule biosynthetic process, ribosome assembly, organelle assembly, non-membrane-bounded organelle assembly, gene expression, organonitrogen compound metabolic process, ribonucleoprotein complex assembly, ribonucleoprotein complex subunit organization, cellular macromolecule metabolic process, and protein metabolic process. Screening revealed that, in addition to metabolic processes and secondary metabolite synthesis, this group also included assembly processes of diverse organelles. These findings suggest that in Group A, plant development is influenced not only through altered metabolic activity but also via regulation of organelle assembly. Moreover, the upregulated anabolism of organic substances such as amides and peptides indicates that the active expression of these compounds may further enhance plant growth and development (Figure 6c).

In Group E (ALL vs. E2), GO enrichment analysis revealed that DEGs were mainly enriched in large ribosomal subunit, cytosolic large ribosomal subunit, ribosomal subunit, cytosolic ribosome, extracellular region, and ribosome within the CC category. These findings indicate that this group may affect plant development by regulating ribosome-related functions, with differential genes also enriched in the extracellular region, likely because ribosome synthesis–related proteins exert different effects extracellularly. In MF, DEGs were significantly enriched in catalytic activity, sulfurtransferase activity, hydrolase activity (acting on glycosyl bonds and hydrolyzing O-glycosyl compounds), and FMN binding. This shows that DEGs were enriched in various enzyme proteins and functions related to signal transduction, potentially influencing plant growth and development by controlling intracellular environmental changes through complex signaling networks. In BP, DEGs were significantly enriched in cellular amide metabolic process, amide biosynthetic process, peptide metabolic process, carboxylic acid metabolic process, oxoacid metabolic process, organic acid metabolic process, translation, peptide biosynthetic process, α-amino acid metabolic process, organonitrogen compound metabolic process, organonitrogen compound biosynthetic process, L-phenylalanine metabolic process, L-phenylalanine catabolic process, erythrose-4-phosphate/phosphoenolpyruvate family amino acid metabolic process, erythrose-4-phosphate/phosphoenolpyruvate family amino acid catabolic process, cellular macromolecule biosynthetic process, aspartate family amino acid biosynthetic process, monocarboxylic acid metabolic process, and small molecule metabolic process. These results indicate that in this group, DEGs were enriched in diverse metabolic processes and the synthesis of secondary metabolites. The upregulated anabolism of organic substances such as amides and peptides suggests that their active expression may contribute to enhanced plant growth and development (Figure 6d).

Finally, enrichment analysis was conducted on Group E (ALL vs. C9). According to the significance ranking of DEGs, only the nucleus was enriched in CC among the top 30 terms, suggesting that DEG enrichment in CC was relatively weak. Moreover, upregulated and downregulated genes in the nucleus exhibited essentially the same state, indicating that this category had a relatively minor impact on plant development. In MF, DEGs were significantly enriched in transferase activity, catalytic activity, hydrolase activity (hydrolyzing O-glycosyl compounds and acting on glycosyl bonds), glutathione transferase activity, quercetin 7-O-glucosyltransferase activity, manganese ion binding, glycosyltransferase activity, galactosyltransferase activity, galactinol–sucrose galactosyltransferase activity, UDP-glucosyltransferase activity, glucosyltransferase activity, iron ion binding, hexosyltransferase activity, NAD^+^ ADP-ribosyltransferase activity, phosphoadenylyl-sulfate reductase (thioredoxin) activity, oxidoreductase activity acting on sulfur group donors with disulfide as acceptor, pentosyltransferase activity, acyltransferase activity (transferring groups other than amino-acyl groups), FMN binding, and ATP-dependent DNA activity. The DEGs enriched in this group included various enzyme proteins, transmembrane transport proteins, and components related to signal transduction. This complex signaling network suggests that differences in plant development between the ALL and C9 groups may result from distinct metabolic and signal transduction pathways. In BP, DEGs were significantly enriched in cell wall macromolecule catabolic process, carbohydrate transmembrane transport, cell wall macromolecule metabolic process, cell wall organization or biogenesis, mitotic chromosome condensation, phototropism, and response to bacterium. These findings indicate that the impact of metabolic processes in this group on plant development may be relatively minor. The most conspicuous differences between ALL and C9 were associated with the expression of genes related to the cell wall (Figure 6e).

Using Group K (ALL vs. CK) as a reference, it was found that the enrichment significance of the CC pathway in the single-strain groups (F, A, E, and C) was lower. This may be because the ALL group contained multiple strains, resulting in fewer differences relative to single-strain groups than to the control. In MF, Groups A2 and E2 exhibited fewer significantly enriched DEGs, suggesting smaller differences in signal network regulation between ALL and these single-strain treatments, similar to the results for Group K. In BP, all groups except Group C enriched more pathways. Therefore, variations in metabolic pathways appear to be the key factors contributing to differences in maize growth and development among the groups.

#### 3.7.2. KEGG Pathway Enrichment Analysis of Differentially Expressed Genes (DEGs)

The maize root transcriptome genes identified were compared with the KEGG database for metabolic pathway analysis. Results showed that DEGs were significantly enriched in multiple metabolic and signal transduction pathways under different strain treatments, involving primary metabolism, secondary metabolism, and plant signal transduction processes.

**Figure 7:**
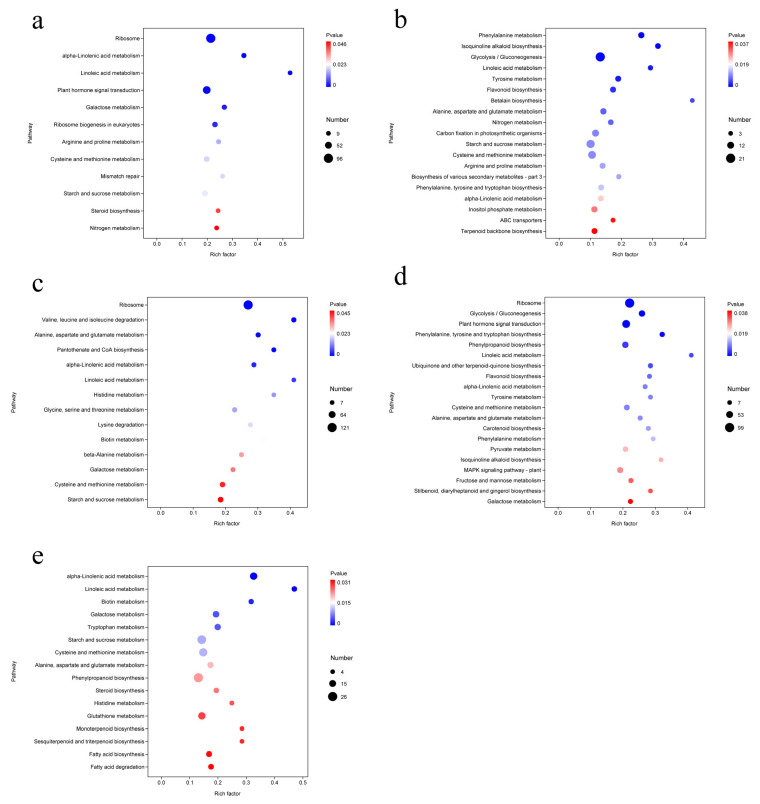
Comparison of KEGG functional annotation results for differentially expressed genes (DEGs) in treatment combinations. (**a**) CK vs. ALL; (**b**) F3 vs. ALL; (**c**) A2 vs. ALL; (**d**) E2 vs. ALL; (**e**) C9 vs. ALL.

Comparative analysis of Group K (ALL vs. CK) revealed that enriched pathways were primarily related to metabolism and genetic information processing. Significantly enriched pathways included ribosome, α-linolenic acid metabolism, linoleic acid metabolism, plant hormone signal transduction, galactose metabolism, ribosome biogenesis in eukaryotes, arginine and proline metabolism, cysteine and methionine metabolism, mismatch repair, starch and sucrose metabolism, steroid biosynthesis, and nitrogen metabolism. Screening indicated that these pathways were associated with three functional categories: metabolic processing, environmental information processing, and genetic information processing. The enrichment of pathways such as lipid metabolism, carbohydrate metabolism, and amino acid metabolism suggests that the ALL treatment enhanced lipid and carbohydrate metabolic activity, thereby supplying sufficient nutrients to support plant growth (Figure 7a).

In Group F (ALL vs. F3), DEGs were mainly enriched in phenylalanine metabolism, isoquinoline alkaloid biosynthesis, glycolysis/gluconeogenesis, linoleic acid metabolism, tyrosine metabolism, flavonoid biosynthesis, betalain biosynthesis, alanine, aspartate and glutamate metabolism, nitrogen metabolism, carbon fixation in photosynthetic organisms, starch and sucrose metabolism, cysteine and methionine metabolism, arginine and proline metabolism, biosynthesis of various secondary metabolites (part 3), phenylalanine, tyrosine and tryptophan biosynthesis, α-linolenic acid metabolism, inositol phosphate metabolism, ABC transporters, and terpenoid backbone biosynthesis. These enriched pathways were related to metabolic processes and environmental information processing but not genetic information processing, indicating minimal genetic differences between the F3 strain and the ALL group. The ALL treatment promoted plant growth by modulating multiple metabolic pathways, including amino acid, lipid, and carbohydrate metabolism (Figure 7b).

In Group A (ALL vs. A2), significantly enriched DEGs, ranked by significance, were ribosome, valine/leucine/isoleucine degradation, alanine/aspartate/glutamate metabolism, pantothenate and CoA biosynthesis, α-linolenic acid metabolism, linoleic acid metabolism, histidine metabolism, glycine/serine/threonine metabolism, lysine degradation, biotin metabolism, β-alanine metabolism, galactose metabolism, cysteine and methionine metabolism, and starch and sucrose metabolism. These pathways fell into the categories of metabolic processing, environmental information processing, and genetic information processing, indicating that the addition of strain A2 still introduced genetic information differences compared with the ALL group, although differences in metabolic pathway enrichment were dominant in this comparison (Figure 7c).

In Group E (ALL vs. E2), significantly enriched DEGs included ribosome, glycolysis/gluconeogenesis, plant hormone signal transduction, phenylalanine/tyrosine/tryptophan biosynthesis, phenylpropanoid biosynthesis, linoleic acid metabolism, ubiquinone and other terpenoid-quinone biosynthesis, flavonoid biosynthesis, α-linolenic acid metabolism, tyrosine metabolism, cysteine and methionine metabolism, alanine/aspartate/glutamate metabolism, carotenoid biosynthesis, phenylalanine metabolism, pyruvate metabolism, isoquinoline alkaloid biosynthesis, MAPK signaling pathway (plant), fructose and mannose metabolism, stilbenoid/diarylheptanoid/gingerol biosynthesis, and galactose metabolism, in descending order of significance. These pathways were associated with metabolic processing, environmental information processing, and genetic information processing. This indicates that the addition of strain E2 still resulted in genetic information differences compared with the ALL group; however, differences in metabolic pathway enrichment remained the dominant feature in this comparison (Figure 7d).

In Group C (ALL vs. C9), significantly enriched DEGs, ranked from highest to lowest, included α-linolenic acid metabolism, linoleic acid metabolism, biotin metabolism, galactose metabolism, tryptophan metabolism, starch and sucrose metabolism, cysteine and methionine metabolism, alanine/aspartate/glutamate metabolism, phenylpropanoid biosynthesis, steroid biosynthesis, histidine metabolism, glutathione metabolism, monoterpenoid biosynthesis, sesquiterpenoid and triterpenoid biosynthesis, fatty acid biosynthesis, and fatty acid degradation. These enriched pathways were associated solely with metabolic processes, further confirming that differences in metabolic pathway enrichment are the key factors contributing to variations in plant growth and development (Figure 7e).

### 3.8. Transcription Factor Analysis

Plant transcription factors are key regulatory proteins that control gene expression by recognizing and binding to specific DNA sequences, thereby activating or inhibiting the transcription of downstream genes. They play essential roles in plant growth, development, responses to environmental stresses, and secondary metabolism.

**Figure 8:**
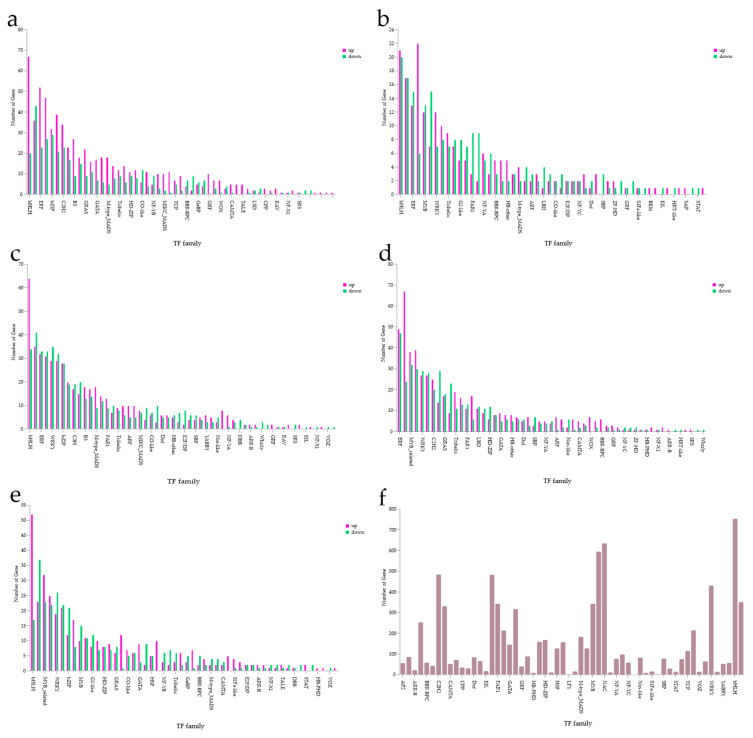
Distribution of transcription factor families. (**a**) CK vs. ALL; (**b**) F3 vs. ALL; (**c**) A2 vs. ALL; (**d**) E2 vs. ALL; (**e**) C9 vs. ALL; (**f**) overall distribution of transcription factor families.

Transcription factors identified through comparison with the Plant Transcription Factor Database were distributed across 29 families (Figure 8f).

In the CK and ALL groups, the 10 families with the largest numbers of transcription factors were bHLH, ERF, bZIP, C2H2, B3, GRAS, GATA, M-type MADS, Trihelix, and HD-ZIP (Figure 8a). In the F3 and ALL groups, the top 10 families were bHLH, ERF, MYB, WRKY, Trihelix, G2-like, FAR1, NF-YA, BBR-BPC, and HB-other (Figure 8b). In the A2 and ALL groups, the top 10 families were bHLH, ERF, WRKY, bZIP, C3H, B3, M-type MADS, FAR1, Trihelix, and ARF (Figure 8c). In the E2 and ALL groups, the top 10 families were ERF, MYB-related, WRKY, C2H2, GRAS, Trihelix, FAR1, LBD, HD-ZIP, and GATA (Figure 8d). In the C9 and ALL groups, the top 10 families were bHLH, MYB-related, WRKY, bZIP, MYB, G2-like, HD-ZIP, GRAS, CO-like, and GATA (Figure 8e). Comprehensive statistics showed that the three largest transcription factor families overall were bHLH, NAC, and MYB-related. The identification of these transcription factors provides a foundation for deeper investigation into the growth-promoting mechanisms of PGPR.

## 4. Discussion

### 4.1. The Effects of PGPR on Maize Phenotypes

PGPR can promote plant growth by solubilizing phosphorus, fixing nitrogen, releasing potassium, enhancing plant enzyme activity, strengthening root systems, facilitating beneficial microorganisms, or suppressing plant pathogens [34]. In this experiment, PGPR were screened from the maize rhizosphere, and strains with strong growth-promoting ability were selected to construct SynComs. Using the non-inoculated PGPR treatment (CK) as the control, five treatments were established: F3, A2, E2, C9, and ALL. Among these, four were single-strain PGPR treatments, and one was the SynComs treatment group (ALL).

During strain screening, some single strains exhibited growth-promoting effects on maize seedlings; however, most strains showed limited effects, with some promoting only root growth or only above-ground growth. Experimental results indicated that maize plant height was significantly increased by five treatments: D4, D10, E4, E2, and E1. The remaining treatments showed differences compared with the control, but these were not significant. Fresh shoot weight was significantly increased by one treatment, E2, while differences observed in other treatments were not significant. The number of root tips was significantly increased by nine treatments: A2, D5, D2, D8, D10, D3, D4, C7, and A7. Other treatments showed differences compared with the control, but these were not significant. For dry weight, root length, and branching, no strain significantly promoted growth, although some treatments showed non-significant differences compared with the control. These results demonstrated that while some PGPR strains in maize roots exerted measurable growth-promoting effects, most strains did not show prominent effects [35].

In this study, inoculation of maize plants with SynComs composed of *Burkholderia* sp. A2, *Pseudomonas* sp. C9, *Curtobacterium pusillum* E2, and *Bacillus velezensis* F3 significantly increased plant height, dry weight, fresh weight, number of branches, and number of root tips. These findings indicate that SynCom inoculation plays a crucial role in promoting maize seedling growth and regulating root morphology. With the exception of slightly lower performance than Group E2 in root system parameters, SynComs were more effective than any single growth-promoting strain. Previous studies have demonstrated that *Burkholderia*, *Pseudomonas*, and *Bacillus velezensis* can promote plant growth, confer resistance to adverse environmental conditions, and inhibit plant diseases when applied individually. For example, *Pseudomonas* sp. has been shown to enhance sugarcane height and increase wheat yield under field conditions [36]. Similarly, *Burkholderia* sp. promotes sustainable rice production by producing indole-3-acetic acid (IAA) and solubilizing phosphates [37]. *Bacillus velezensis* contributes to biological control by inhibiting plant pathogens on leaves and roots, thereby supporting plant growth [38].

Overall, inoculation of maize seedlings with SynComs led to significant changes in biomass and root morphology. It is likely that SynComs altered the composition, diversity, and function of the rhizosphere microbial community, thereby promoting plant growth and development [39,40]. Furthermore, the multiple strains comprising the SynCom are speculated to exert complementary functional effects.

### 4.2. The Impact of SynComs on Maize Transcriptomics Analysis

Transcriptome analysis provides valuable insights into how plants respond to different microbial community treatments by examining overall patterns of gene expression. Although substantial transcriptomic information has been generated in studies on maize [41], research specifically addressing transcriptome sequencing in the context of growth-promoting mechanisms remains limited.

Transcriptome sequencing was performed on maize roots treated with SynComs and single strains, followed by a systematic evaluation of the sequencing results. All sequencing quality parameters were within optimal ranges: clean reads (≥97.05%), GC content (48.14–52.13%), Q20 (≥98.87%), and Q30 (≥95.71%). These data verified that quality parameters complied with technical standards for plant RNA sequencing and were suitable for subsequent gene expression analysis [42].

To ensure data reproducibility and rational sample distribution, sample correlation analysis, gene expression boxplots, and principal component analysis (PCA) were performed. Boxplot analysis revealed that FPKM values of all samples were uniformly distributed on the log10 scale, with no abnormal values or deviations. This supported the effectiveness of FPKM normalization in minimizing the influence of sequencing depth variation and sample heterogeneity. PCA showed PC1 (45.3%) and PC2 (28.4%), demonstrating strong reproducibility within sample groups. Distinct clusters among different treatments reflected differences in transcription profiles, confirming that transcriptome-level differentiation corresponded with treatment effects. These results suggest that transcriptomic profiles can successfully distinguish maize roots with different metabolic characteristics, consistent with findings from other studies [43].

To systematically analyze transcriptional differences between SynComs and single-strain groups, GO functional classification and KEGG pathway enrichment analyses were conducted. In GO enrichment, DEGs were significantly associated with ribosome, cellular amide biosynthetic and metabolic processes, flavonoid biosynthetic and metabolic processes, peptide biosynthetic and metabolic processes, biosynthetic processes, transferase activity, catalytic activity, and manganese and iron ion binding. This indicates that multiple pathways may jointly regulate maize seedling growth in response to microbial treatments. Previous studies have shown that certain maize genes encode proteins that influence seedling growth by modulating ribosome biosynthesis [44]. Similarly, the roles of amides, flavonoids, and peptide compounds in plant cells have been validated. Flavonoids regulate protein activity, thereby influencing plant cell growth and activation [45]. Peptide compounds have demonstrated stronger growth-promoting effects compared with plant hormones [46]. Some amide compounds serve as precursors of essential coenzymes, thereby supporting plant growth [47]. The contributions of transferases, catalytic functions, and iron and manganese ion activity to plant development have also been confirmed in various studies [48,49,50].

In KEGG enrichment, eight metabolic pathways were significantly associated with DEGs, including carbohydrate metabolism, amino acid metabolism, lipid metabolism, translation, and the biosynthesis of other secondary metabolites. Carbohydrates such as starch, cellulose, and sugar alcohols participate in carbohydrate metabolism [51]. Treatments with different microbial communities may alter plant growth by influencing the synthesis of these carbohydrates. Plants interact with microorganisms through amino acid metabolism, which provides signaling molecules, generates protective compounds, and supplies nutrients [52]. For example, aromatic amino acids synthesized in the cytoplasm via the shikimic acid pathway interact with microorganisms, including in the production of protective phytoalexins [53,54]. Lipid metabolism, a fundamental pathway in plants, encompasses membrane lipid metabolism and fatty acid metabolism, both of which are essential for growth, cold resistance, and hormone regulation [55]. Studies also indicate that strains can respond to maize root exudates and alter amino acid, carbohydrate, and lipid metabolism pathways by regulating intracellular ROS levels [56]. Furthermore, the addition of SynComs directly or indirectly regulates the expression of genes associated with translation, cofactors and vitamins, energy metabolism, and the biosynthesis of other secondary metabolites. Collectively, these regulations enhance maize seedling growth by influencing development, modifying responses to environmental stimuli, altering primary metabolism, and stimulating the synthesis of diverse secondary metabolites [57,58,59].

Analysis of transcription factor families containing differentially expressed genes in maize roots revealed that transcription factors were primarily distributed among bHLH, NAC, MYB-related, C2H2, ERF, and WRKY families, all exhibiting mixed regulatory modes. Previous studies have demonstrated that several transcription factor families are closely linked to plant growth and development. The bHLH gene family, for example, is widely present in plants and strongly associated with normal growth and development, including roles in stress signal transduction and diverse regulatory functions in gene expression [60,61]. As one of the largest protein families in plants, MYB participates in both primary and secondary metabolism and contributes to responses against various stresses, with an especially critical role in stress adaptation [62]. The NAC transcription factor family enhances plant immunity through multiple signaling pathways and is essential in regulating disease resistance [63]. Thus, the upregulation and downregulation of these transcription factor families help explain differences in maize growth across treatment groups. Collectively, these transcription factors provide important clues for understanding the gene regulatory network in maize roots and highlight that distinct transcription factor families exhibit diverse regulatory patterns in response to treatments with different strains.

## 5. Conclusions

This study screened PGPR from maize roots and conducted growth-promoting experiments to compare phenotypic differences in maize roots treated with different strains and SynComs. In addition, systematic cluster analysis and differential expression profiling based on RNA-seq were used to comprehensively examine transcriptome regulation and metabolic pathways in maize roots under different treatments. The results demonstrated that certain PGPR with growth-promoting effects could be identified from maize roots, and that SynComs exerted stronger growth-promoting effects on maize roots than single strains. The complementary functions of the individual strains comprising the SynCom were reflected in its enhanced overall effects.

GO functional classification and KEGG pathway enrichment analyses indicated that SynComs treatment regulated maize seedling root growth through multiple pathways, including carbohydrate metabolism, amino acid metabolism, lipid metabolism, translation, and the biosynthesis of other secondary metabolites. Most of the enriched genes were associated with cellular metabolism, suggesting that strains promote maize seedling growth through diverse metabolic processes.

In conclusion, this study provides insights into rhizosphere–plant interactions at the transcriptional regulation level, establishes a solid scientific basis for further improving maize seedling growth, and presents a comprehensive molecular framework for understanding the growth-promoting mechanisms of strains and SynComs in maize roots. Although SynComs were shown to promote maize seedling growth by regulating multiple molecular-level metabolic pathways, subsequent field experiments are required. Furthermore, the specific roles of individual metabolic genes must be clarified at the molecular level, and their relative contributions to growth should be evaluated.

## Figures and Tables

**Table 1:** Selected strains and their effects.

	1	2	3	4
Strain	F3	A2	E2	C9
inductive site	root system and aboveground parts	root system	aboveground parts	aboveground parts

**Table 2:** Assessment and Statistics of RNA-Seq Sequencing Data.

Sample Name	Raw Reads	Raw Bases	Clean Reads	Clean Reads (%)	GC (%)	N (%)	Q20 (%)	Q30 (%)
CK_1	40,027,872	6,044,208,672	39,653,424	99.06	48.14	0.002272	99.25	96.93
CK_2	43,879,234	6,625,764,334	43,281,774	98.64	48.20	0.005105	99.02	96.19
CK_3	43,883,538	6,626,414,238	43,434,494	98.98	48.97	0.005551	99.11	96.33
F3_1	39,906,264	6,025,845,864	39,410,152	98.76	50.42	0.022185	99.03	96.09
F3_2	38,521,754	5,816,784,854	37,899,476	98.38	50.57	0.022312	98.90	95.71
F3_3	47,075,358	7,108,379,058	46,369,904	98.50	50.31	0.022086	98.94	95.90
A2_1	41,600,470	6,281,670,970	40,996,692	98.55	51.40	0.022259	98.93	95.84
A2_2	40,234,790	6,075,453,290	39,644,330	98.53	51.26	0.020882	98.96	96.04
A2_3	58,848,234	8,886,083,334	57,305,442	97.38	52.13	0.020966	98.87	95.83
E2_1	63,049,274	9,520,440,374	62,450,540	99.05	49.58	0.002383	99.33	97.16
E2_2	41,202,820	6,221,625,820	39,985,792	97.05	50.31	0.002375	99.17	96.57
E2_3	63,041,154	9,519,214,254	61,614,760	97.74	49.87	0.020898	98.94	95.98
C9_1	74,226,278	11,208,167,978	73,169,600	98.58	48.34	0.021040	99.00	96.25
C9_2	51,605,820	7,792,478,820	51,133,510	99.08	48.45	0.002445	99.32	97.14
C9_3	66,848,094	10,094,062,194	65,892,666	98.57	49.34	0.020993	99.00	96.20
All_1	43,758,280	6,607,500,280	43,135,644	98.58	49.97	0.021116	99.05	96.41
All_2	55,023,878	8,308,605,578	53,850,038	97.87	50.51	0.011873	99.04	96.07
All_3	47,243,956	7,133,837,356	46,829,536	99.12	50.00	0.002400	99.37	97.26

## Data Availability

The original contributions presented in this study are included within the article. Further inquiries can be directed to the corresponding authors.

## Abbreviations

The following abbreviations are used in this manuscript:

SynCom	Synthetic microbial community
CK	control group
